# Amyloid-β Peptide on Sialyl-Lewis^X^-Selectin-Mediated Membrane Tether Mechanics at the Cerebral Endothelial Cell Surface

**DOI:** 10.1371/journal.pone.0060972

**Published:** 2013-04-12

**Authors:** Sholpan Askarova, Zhe Sun, Grace Y. Sun, Gerald A. Meininger, James C-M. Lee

**Affiliations:** 1 Department of Biological Engineering, University of Missouri, Columbia, Missouri, United States of America; 2 Dalton Cardiovascular Research Center, University of Missouri, Columbia, Missouri, United States of America; 3 Department of Biochemistry, University of Missouri, Columbia, Missouri, United States of America; 4 Department of Medical Pharmacology and Physiology, University of Missouri, Columbia, Missouri, United States of America; 5 Department of Biomedical Engineering, Cell Technologies, and Transplantation, Center for Life Sciences, Nazarbayev University, Astana, Kazakhstan; Dalhousie University, Canada

## Abstract

Increased deposition of amyloid-β peptide (Aβ) at the cerebral endothelial cell (CEC) surface has been implicated in enhancement of transmigration of monocytes across the brain blood barrier (BBB) in Alzheimer's disease (AD). In this study, quantitative immunofluorescence microscopy (QIM) and atomic force microscopy (AFM) with cantilevers biofunctionalized by sialyl-Lewis^x^ (sLe^x^) were employed to investigate Aβ-altered mechanics of membrane tethers formed by bonding between sLe^x^ and p-selectin at the CEC surface, the initial mechanical step governing the transmigration of monocytes. QIM results indicated the ability for Aβ to increase p-selectin expression at the cell surface and promote actin polymerization in both bEND3 cells (immortalized mouse CECs) and human primary CECs. AFM data also showed the ability for Aβ to increase cell stiffness and adhesion probability in bEND3 cells. On the contrary, Aβ lowered the overall force of membrane tether formation (*F_mtf_*), and produced a bimodal population of *F_mtf_*, suggesting subcellular mechanical alterations in membrane tethering. The lower *F_mtf_* population was similar to the results obtained from cells treated with an F-actin-disrupting drug, latrunculin A. Indeed, AFM results also showed that both Aβ and latrunculin A decreased membrane stiffness, suggesting a lower membrane-cytoskeleton adhesion, a factor resulting in lower *F_mtf_*. In addition, these cerebral endothelial alterations induced by Aβ were abrogated by lovastatin, consistent with its anti-inflammatory effects. In sum, these results demonstrated the ability for Aβ to enhance p-selectin expression at the CEC surface and induce cytoskeleton reorganization, which in turn, resulted in changes in membrane-cytoskeleton adhesion and membrane tethering, mechanical factors important in transmigration of monocytes through the BBB.

## Introduction

Alzheimer's disease (AD) is the most prevalent age-related neurodegenerative disease affecting higher cognitive functions, learning, and memory of millions people worldwide. It is the most common cause of dementia and the sixth-leading cause of death among people aged 65 and older. Increased deposition of amyloid β peptide (Aβ) together with the increased numbers of activated microglial cells in the parenchyma, and monocytes in the vessel wall of AD brain have been observed [1], [2]. Recent epidemiological and laboratory studies have indicated the important role of cerebral vascular factors in the progression of AD [3], [4]. Peripheral monocytes have been shown to migrate across the blood-brain barrier (BBB) and differentiate into microglia within the brain parenchyma [5]. *In vitro* studies have provided evidence demonstrating that Aβ deposition at the endothelial cell layer enhances the transmigration of monocytes [6]–[8]. Increased transmigration of monocytes into brains is thought to drive the disease progression towards exacerbation of the oxidative and inflammatory conditions characteristic of the AD brain.

Transmigration of monocytes is a sequential process with three distinct adhesive events: 1) capture, tethering and rolling; 2) firm adhesion and arrest; and 3) crawling on the endothelial surface to find an intercellular junction for transmigration to the target tissue. Primary capture by the endothelium and rolling are mediated by tethering to selectins and selectin ligands [9], [10]. Selectins belong to the type I transmembrane cell adhesion molecule family and are comprised of three members, namely, P, E, and L-selectins. P-and E-selectin are expressed on the endothelial cell surface upon exposure to different pro-inflammatory agents such as TNF-α, interleukin, and lipopolysaccharide. Their roles in immune cell rolling vary depending on the particular stimuli and the type of tissue. The physiological ligands for selectins are glycoproteins, including P-selectin glycoprotein ligand 1(PSGL-1), E-selectin ligand 1 (ESL-1), and CD34. All these ligands carry conjugated carbohydrate sialyl Lewis^x^ (sLe^x^) as an active binding site [11], [12].

**Figure 1:**
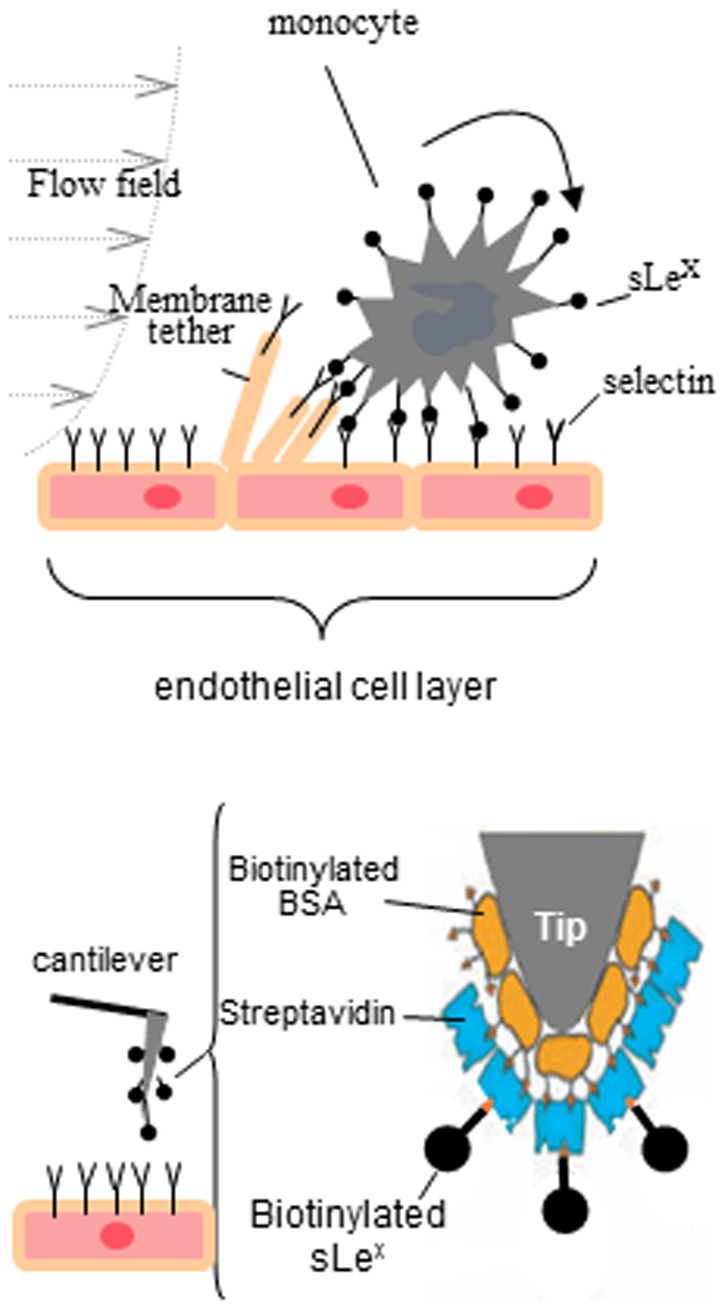
Schematic descriptions of membrane tether formation and biofunctionalization for the AFM cantilever tip. Membrane tether formation mediated by sLe^x^-selectin bonding during a monocyte rolling on the endothelial layer (*upper*) and the strategy using AFM cantilever tips bio-functionalized by sLe^x^ to characterize the mechanics of membrane tether adhesion (*lower*). (modified from Yves F. Dufrêne, 2008).

It is also important to note that cell-cell adhesion is governed by the expression of adhesion molecules and their ligands as well as the mechanical properties of the cells and cell membranes [13]–[15]. Since the transmigration of monocytes across the BBB is both a mechanical and biochemical process, the expression of adhesion molecules and mechanical properties of endothelial cells are critical factors that require investigation. In this study, we applied AFM with cantilever tips bio-functionalized by sLe^x^ (Fig. 1) in combination with quantitative immunofluorescence microscopy (QIM) to study the direct effects of Aβ oligomers on selectin expression, actin polymerization, and mechanical and adhesion properties in cerebral endothelial cells. In addition, we examined if lovastatin, a cholesterol-lowering drug, attenuates Aβ effects.

## Materials and Methods

### Reagents

Aβ_1–42_ from American Peptides were prepared by diluting 5 mM Aβ_1–42_ in DMSO to 100 µM in ice-cold culture Ham's medium and incubating at 4°C for 24 h. Lovastatin (Calbiochem) was prepared following the manufacturer's instruction. Briefly, Lovastatin was converted into its active form by dissolving it in absolute ethanol (20 µg/µl lovastatin) followed by the addition of 1N NaOH (to final concentration of 0.45 M). This solution could be stored at −20°C until used. The solution was neutralized (pH 7.2) with 1 N HCl immediately prior to the use. Latrunculin A (Sigma) was dissolved in DMSO and then diluted in cell culture medium to final concentration of 1 µM.

### Cell culture

Mouse immortalized cerebral endothelial cells (bEnd3 line) were from ATCC (Manassas, VA, USA), and primary human cerebral endothelial cells (CEC) were from ScienCell Research Laboratories (Carlsbad, CA, USA). Cells were cultured in DMEM with 10% PBS and 1% antibiotic/antimycotic and maintained in humidified 5% CO_2_ incubator at 37°C.

### Cell treatments

To demonstrate the effects of Aβ oligomers on CECs, cells were treated with two different concentrations of Aβ oligomers (0.5 µM and 1 µM) for 20 min prior to further characterizations. To test if statin was capable of counteracting the effects of Aβ, cells were pre-treated with Lovastatin (20 µM) for 1 h, followed by Aβ treatment (1 µM). For a positive control of inflammatory responses, CECs were treated with histamine (10 µM for 20 min). To demonstrate the role of cytoskeletal organization, cells were incubated with 1 µM Latrunculin A (an actin polymerization inhibitor) for 30 min before characterizations.

### Immunofluorescent labeling

Cells were grown on cover slips until confluent. After treatments, cells were fixed immediately using 3.7% paraformaldehyde solution for 30 min. To block non-specific binding, 5% BSA in PBS was applied to cells for 1 h. P- or E-selectins at the cell surface were labeled with its primary antibody (R&D systems) without permeabilization at 4°C overnight, followed by goat Alexa Fluor 594 anti-rat secondary antibody (Invitrogen) at 25°C for 1 h. To confirm the specificity of the selectin primary antibodies, labeling by secondary antibodies alone did not show immuno-staining in the absence of the primary antibody. For F-actin labeling, cells were permeablized by 0.1% Triton X-100 in PBS for 5 min and incubated with Oregon-green phalloidin (250 nM) (Invitrogen) in PBS with 1% BSA at 25°C for 1 h.

### Quantitative Immunofluorescence microscopy (QIM)

Bright-field illumination and fluorescence microscopy were performed with Nikon TE-2000 U fluorescence microscope and 40×, NA 0.95 objective. Images were acquired using a cooled-CCD camera controlled with a computer that runs MetaView imaging software. The typical exposure time for fluorescence image acquisition was 400 msec. Background subtraction was performed for all images prior to analysis. Actin polymerization was quantified by calculating the intensity of Oregon Green-phalloidin-labeled F-actin per cell area. The intensity was then normalized by the intensity of the labeled F-actin in control cells (without any treatment). A similar approach was applied to quantify the relative expression of P- and E- selectins. A total 300 images were analyzed.

### Atomic Force Microscopy

A Bioscope system from Veeco, Inc. equipped with Nanoscope IVa controller and Nanoscope 5.12 software and mounted on the top of an Olympus IX81 microscope (Olympus) was used to perform mechanical and adhesive measurements. All force curves were processed with the NForceR software (Copyright October 10, 2006; Registration Number TXu1-328-659, Cardiovascular Research Institute, Texas A&M University System).

### Bio-functionalization of the AFM cantilever tips

We adapted the procedure used by Micic (1999) [16] to functionalize Si_3_N_4_ cantilevers with avidin, followed by incubation with biotinylated-sLe^x^ to further functionalize cantilevers with sLe^x^ coated surface (Fig. 1, *lower*). Briefly, after a 5-min wash in acetone followed by UV irradiation for 15 min, triangular silicon nitride cantilevers (Veeco, Texas) were immersed in 50 µl of biotin-BSA (Sigma; 0.5 mg/ml in 100 mM NaHCO_3_) and incubated overnight in 37°C humid chamber. The biotin-BSA coated cantilevers were rinsed several times with PBS and fixed in 20% glutaraldehyde for 30 min, followed by incubation in the solution containing avidin (Neutravidin; Pierce, 0.5 mg/ml in 100 mM NaHCO_3_). Avidin functionalized cantilevers were washed and then incubated in the 0.5 µg/ml biotin-sLe^x^ solution (Glycotech, Gaithersburg, MD). At the loading rate ∼400 pN/s, the unbinding force of avidin-biotin is ∼200 pN [17], which is much greater than that of p-selectin-sLe^x^ (∼50 pN) [12]. Therefore, the biotin-avidin bond was assumed not to break during our experiments.

### Measurements for cell adhesion probability, cell and membrane stiffness, and force for membrane tether formation (*F_mtf_*)

**Figure 2:**
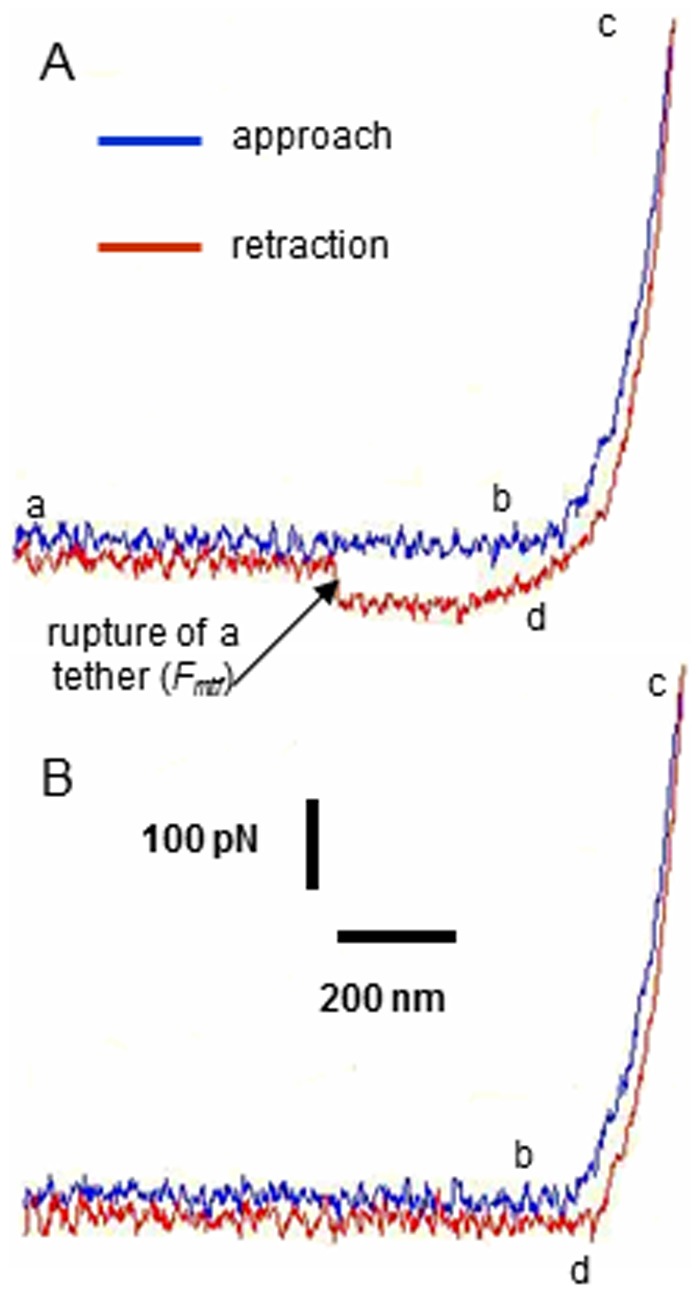
Typical force curves obtained from AFM measurement. (*A*) Approach and retraction force curves with adhesion; and (*B*) without adhesion. The cantilever approaches (a to b), touches (b), makes indentation (b to c) and retracts (d) from the cell. Force of membrane tether formation (*F_mtf_*) was measured at the sudden drop of force when a rupture of a membrane tether occurred (denoted by an arrow).

The AFM was set to operate in force mode, and the piezotransducer (PZT) was set to drive the cantilever to approach, touch, make an indentation of the cell, and retract from the cell over a predefined distance in the optical axis perpendicular to the cell surface, which could be identified from the force curve (Fig. 2). The force curve was generated from the recorded vertical-axis movement of the PZT and the deflection of the cantilever with a known dimension (320 µm in length and 22 µm width with a pyramidal half-angle of 35^0^) and a spring constant of ∼12±3 pN/nm measured by the thermal noise method. Fig. 2A shows a typical force curve with an adhesion event. The sudden release of force occurred at the rupture of a membrane tether was used as a measure of *F_mtf_* through the bonding between p-selectin and sLe^x^ calculated by multiplying the spring constant of the cantilever with the deflection height associated with a membrane tether rupture (Fig. 2A). Since adhesion events sometimes did not occur in some force curves (Fig. 2B), adhesion probability was calculated by dividing the number of curves with adhesion events by the total number of curves. To measure the stiffness of the cell, the part of the AFM force curve representing cell indentation was fitted by the Hertz model: F = (2Eδ^2^)/ [π (1-ν^2^) tan α] to calculate E, the Young's modulus (i.e. the stiffness) of the cell, where F is the force, δ the indentation, ν the Poisson ratio of the cell, assumed to be 0.5, and α the half-operating angle of the indenting cone. To measure membrane stiffness, *ΔF*/(the depth of indentation) are calculated within 5 nm indentation at the cell surface (i.e. *ΔF*/5 nm). The loading and unloading speeds of labeled cantilever tips were 1 µm/s, contact time - 0.2 sec. In this study, at least 2000 curves were analyzed and reported for each experimental group.

## Results

### Oligomeric Aβ_1–42_ enhanced P-selectin expression at the CEC surface

**Figure 3:**
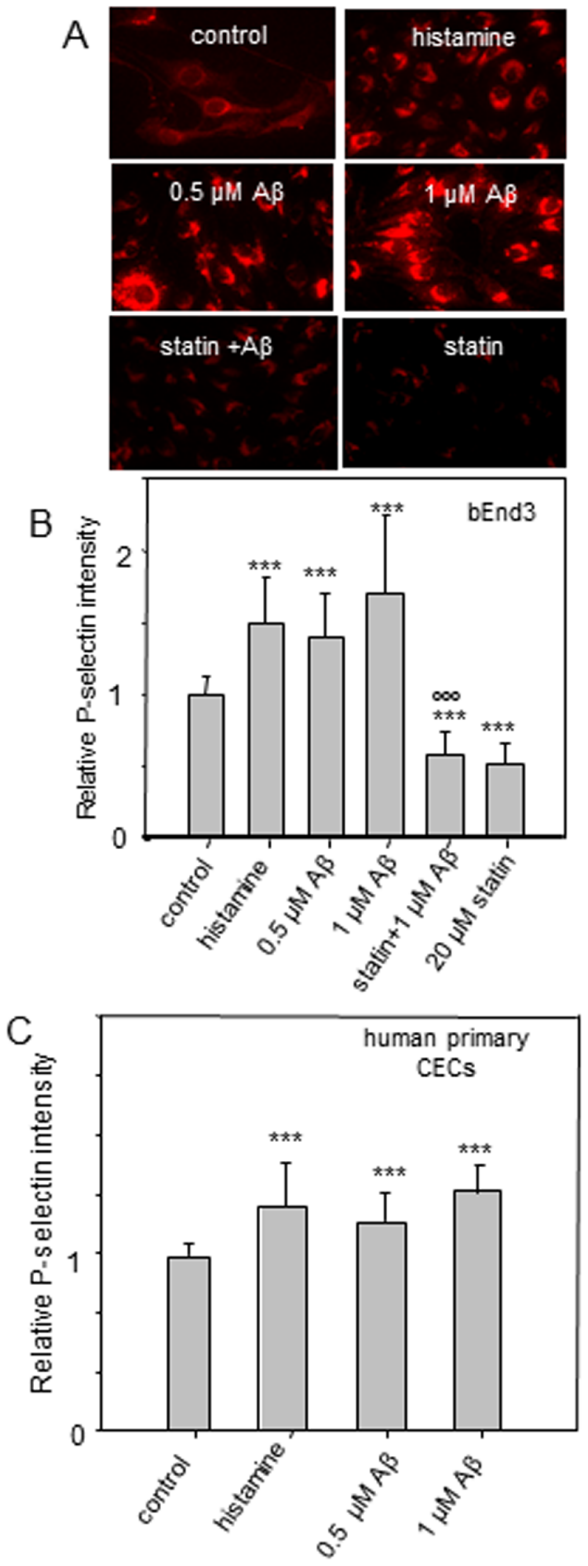
Effects of Aβ, histamine, and lovastatin on P-selectin expression at the CEC surface. (*A*) Fluorescent micrographs of fluorescently-labeled P-selectin at the bEnd3 cells. (*B*) Relative P-selectin intensity at the bEnd3 cell surface and (*C*) the human primary CEC surface. ***p≤0.001, **p≤0.01 compare to the control; °°° p≤0.001 compare to the Aβ (1 µM) treatment group.

To investigate the effects of Aβ_1–42_ oligomers on the expression of P-selectin at the CEC surface, quantitative immunofluorescence microscopy (QIM) of P-selectin was performed without cell permeabilization in the immunostaining procedure. Consistent with the notion that Aβ stimulates CECs for cellular adhesion and transmigration [18], Aβ_1–42_ increased P-selectin expression at the surface of bEnd3 cells by 35–60% (Fig. 3B) and primary human CECs by 10–22% (Fig. 3C). Since histamine is known for its ability to increase P-selectin at the endothelial surface [15], [19], [20], results with histamine were used as a positive control (Fig. 3B). Previous study also reported the ability of lovastatin to abrogate histamine-induced increase in P-selectin expression at the endothelial surface [21]. In this study, lovastatin also suppressed Aβ_1–42_-induced increase in P-selectin expression at the CEC surface (Fig. 3B).

### Aβ_1–42_ enhanced actin polymerization

**Figure 4:**
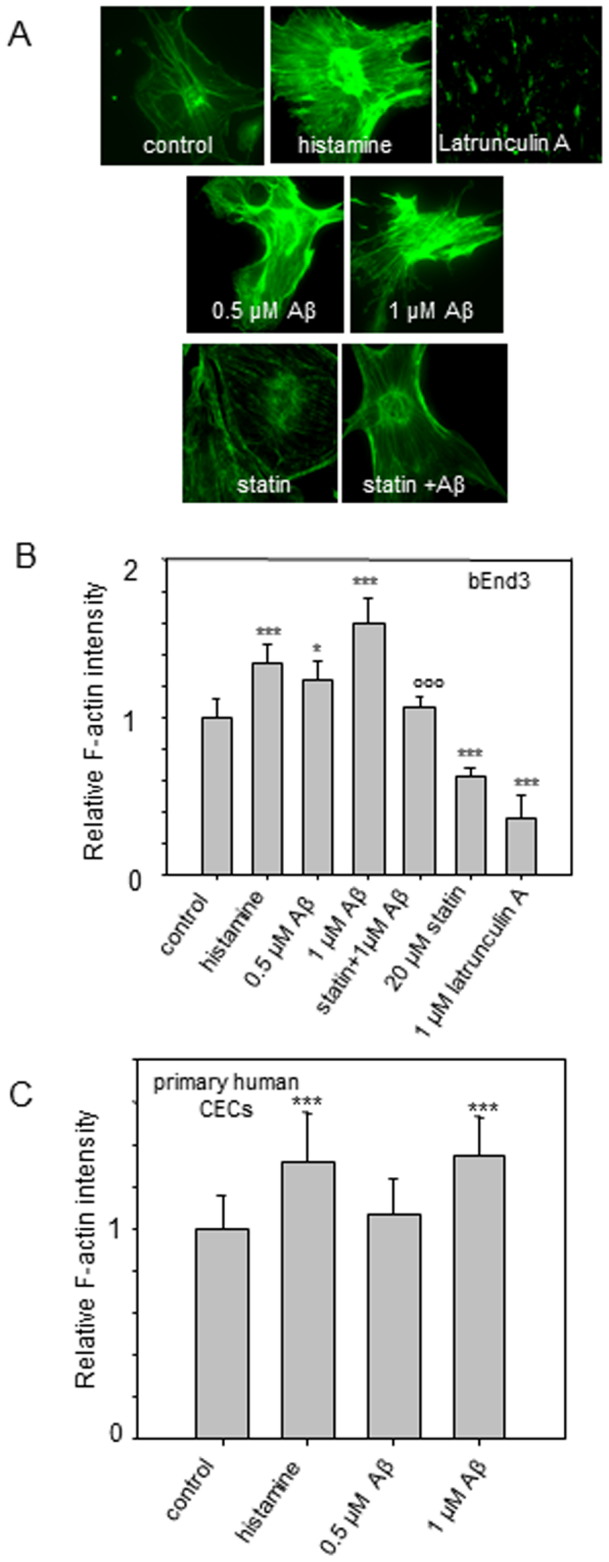
Effects of Aβ, and histamine on actin polymerization in CECs. (*A*) Fluorescent micrographs of Oregon-green phalloidin-labeled F-actin in bEnd3 cells. (*B*) Relative F-actin intensity in bEnd3 cells and (*C*) primary human CECs. ***p≤0.001, ** p≤0.01, * p≤0.05 compare to the control; °°° p≤0.001 compare to the Aβ (1 µM) treatment group.

Aβ_1–42_ has been reported to enhance actin polymerization and induce actin stress fiber formation in neuronal cells. [22], [23]. Since the organization of cytoskeleton governs cell mechanics and adhesion, we employed QIM of F-actin labeled with Oregon green phalloidin to quantify actin polymerization induced by Aβ_1–42_ in CECs. As shown in Fig. 4A, B and C, QIM data showed that Aβ_1–42_ and histamine promoted actin polymerization in both primary human CECs and bEnd3 cells. In contrast, significant reduction of actin intensity was observed when cells were treated with latrunculin A, an F-actin disruptive drug (Fig. 4A). Lovastatin also decreased actin polymerization (Fig. 4B), but unlike latrunculin, lovastatin did not cause a dramatic disorganization of the cytoskeleton structure (Fig. 4A).

### Adhesion probability and molecular specificity of adhesion

**Figure 5:**
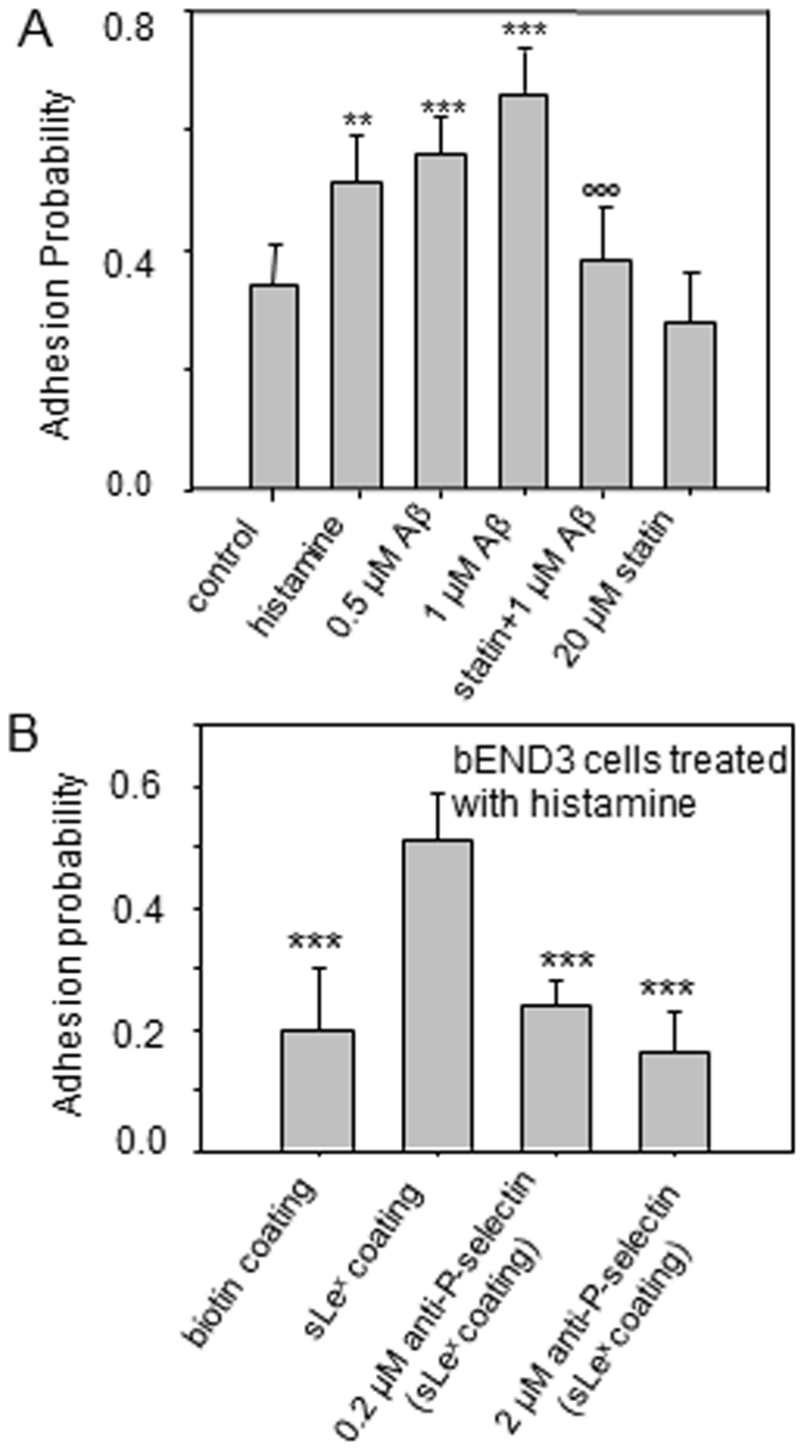
Effects of Aβ, histamine, and lovastatin on adhesion probability at the bEND3 cell surface with AFM cantilever tips biofunctionalized by conjugating sLe^x^ at their surface. (*A*) Adhesion probability was measured for cells treated with histamine, Aβ, lovastatin and Aβ and lovastatin alone. Adhesion probability was calculated by normalizing the number of force curves with adhesion events by the total number of force curves. ***p≤0.001, **p≤0.01 compare to the control; °°°p≤0.001 compare to the Aβ (1 µM) treatment group. (*B*) bEND3 was treated with histamine, and adhesion probability was measured. A highest adhesion probability was obtained for the cantilever coated with sLe^x^; whereas lower adhesion probabilities were obtained for the cantilever coated with biotin only, and cells treated with antibody of P-selectin, indicating that *F_mtf_* measured in this study are highly molecularly specific through sLe^x^-P-selectin bonding. ***p≤0.001compare to the sLe^x^ coating group.

Consistent with QIM results showing that both histamine and Aβ_42_ increased P-selectin at the CEC surface (Fig. 3), AFM data demonstrated that adhesion probability increased upon Aβ_42_ or histamine treatment (0.34±0.07 for control; 0.51±0.08 for histamine; 0.55±0.06 and 0.66±0.07 for 0.5 µM and 1 µM of Aβ_42_ respectively), and inhibited after lovastatin treatment (0.28±0.08) (Fig. 5A).

To address whether these adhesion events are specific through bonding with the selectin-sLe^x^ interactions, AFM data demonstrated that in cells pretreated with histamine, the probability of adhesion for cantilevers coated with only biotin was at least twofold lower as compared to those coated with sLe^x^ (Fig. 5B). Lower adhesion probabilities were also obtained when P-selectin at the cell surface was blocked with its antibodies (Fig. 5B). These results indicated that adhesion events and membrane tether formation in this study are facilitated by specific molecular interactions between p-selectin and sLe^x^.

### Cell stiffness characterized by AFM

**Figure 6:**
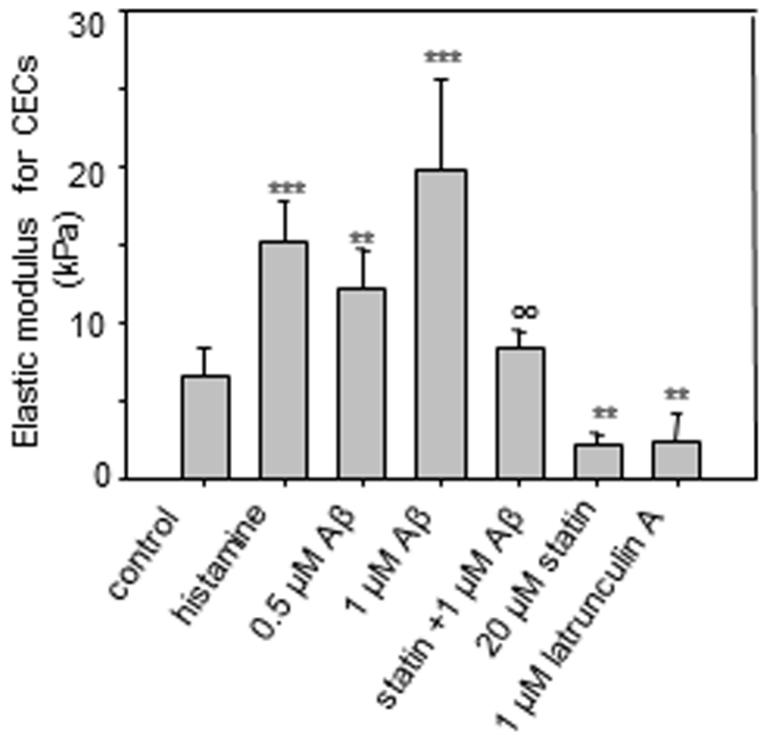
Cell stiffness. (*A*) Cell stiffness (Elastic modulus) for bEND3 cells treated with histamine, Aβ, lovastatin and Aβ, lovastatin alone, and latrunculin A. The elastic modulus was calculated by fitting the cell indentation part of the force curves with Hertz model. ***p≤0.001, **p≤0.01 compare to the control; °°p≤0.01compare to the Aβ (1 µM) treatment group.

The part of the AFM force curves representing cell indentation was used to measure the stiffness of the cells. As the cantilever tip of the AFM approached and made a contact at the endothelial surface (b in Fig. 2A), continued approach makes an indentation (c in Fig. 2A) at the cell surface. The relationship between the force applied to make the indentation and the position of the cantilever was recorded and fitted with Hertz model to calculate the elastic modulus, which characterized the mechanical stiffness and had a value 6.8±1.9 kPa for control CECs (Fig. 6). Both Aβ_42_ and histamine had a dramatic effect on CECs stiffness, showing increases to 15.1±2.7 kPa, 12.2±2.6 kPa, 19.9±5.8 kPa for histamine, 0.5 µM and 1 µM of Aβ_42_, respectively. In contrast, lovastatin and latrunculin A showed an opposite effect and significantly decreased stiffness of the cells as compared to the control (2.3±0.7 kPa and 2.4±1.8 kPa for lovastatin and latrunculin). At the same time, stiffness of the cells was not significantly changed if lovastatin was applied prior to treatment with Aβ_42_ (8.4±1.1 kPa).

### Force of membrane tether formation characterized by AFM

To study the effects of Aβ_42_ oligomers on the force of membrane tether formation (*F_mtf_*) mediated by sLe^x^-selectin bonding, AFM with cantilever tips bio-functionalized by sLe^x^ were applied (Fig. 1). In the force mode, the piezotransducer (PZT) was set to drive the cantilever to approach, touch, make an indentation on the cell, and retract from the cell. As the cantilever was retracted from the cell, the surface of the cell was lifted up and formed membrane tethers from the cantilever tip if adhesion between the cell surface and cantilever tip had occurred [24]. As the cantilever moved further away from the cell, the rupture of tether was detected; and the force required to maintain a membrane tether (i.e. force of membrane tether formation, *F_mtf_*) was recorded with a fixed retraction speed (1 µm/s). The adhesion ruptures were detected in the retraction part of force curves as evident by a sudden change in force acting on the cantilever (Fig. 2A). Retraction curves without adhesion did not exhibit these rupture events (Fig. 2B).

**Figure 7:**
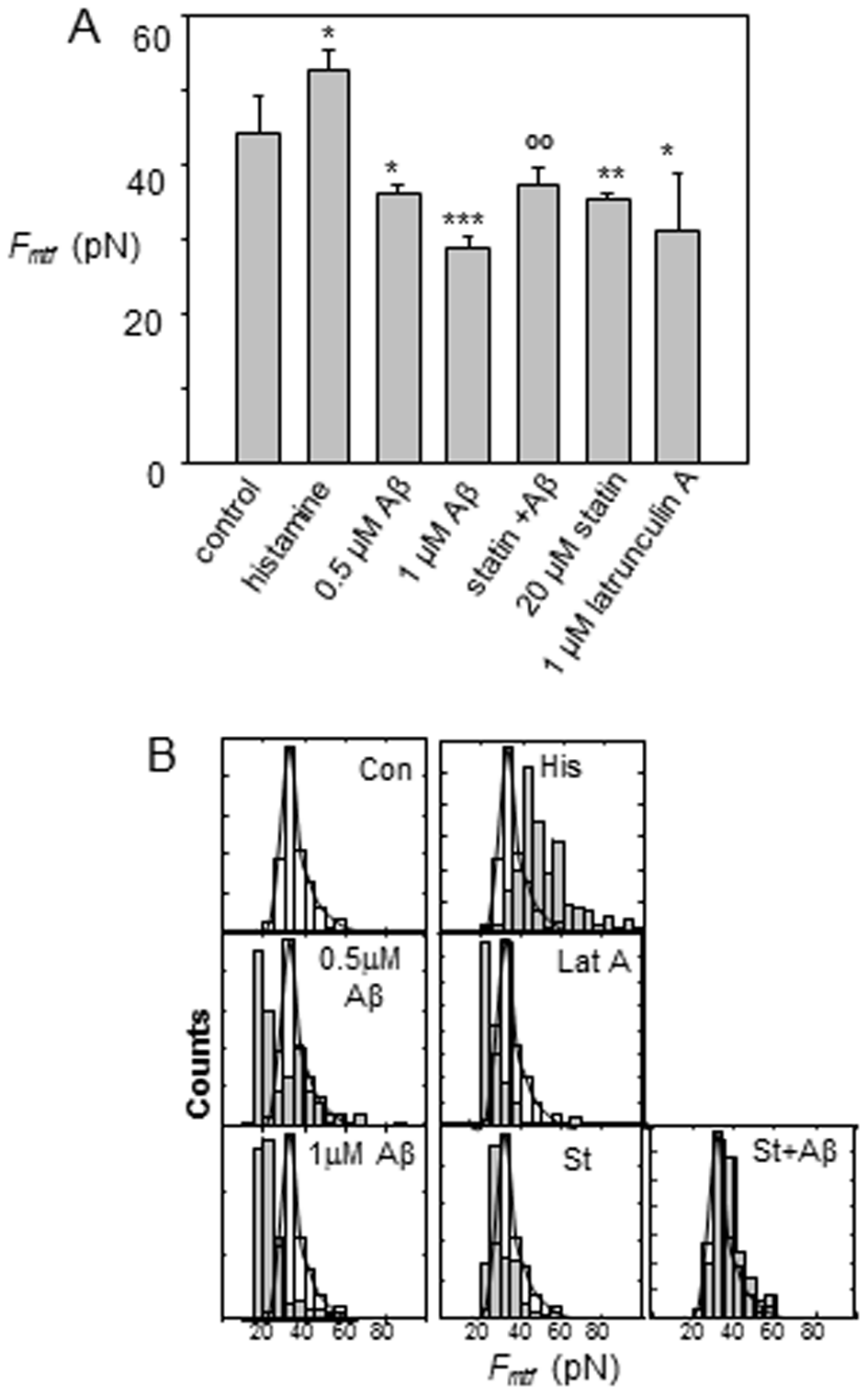
Aβ on force of membrane tether formation (*F_mtf_*) mediated by sLe^x^-selectin bonding. (A) *F_mtf_* was measured at the sudden drop of force when a rupture of a membrane tether occurred (Fig. 2A). A bar graph summarizes *F_mtf_* measured for cells treated with histamine, Aβ, lovastatin and latrunculin A. ***p≤0.001, **p≤0.01, *p≤0.05 compare to the control; °°p≤0.01 compare to the Aβ (1 µM) treatment group. (*B*) The distributions of *F_mtf_* were plotted for different experimental groups. *F_mtf_* distribution for the control group is represented in unfilled bars, and superimposed with other experimental groups represented in grey bars for comparison.

Fig. 7 represents the *F_mtf_* measurement for an untreated (control) and experimental groups of mouse CECs. The average *F_mtf_* was 44±5 pN for control CECs. According to the theory of membrane-cytoskeleton adhesion [25], Aβ-induced actin polymerization as shown in Fig. 4 may increase membrane-cytoskeleton adhesion resulting in a greater *F_mtf_*. Surprisingly, opposite to results with histamine, *F_mtf_* measured for cells treated with 1 µM and 0.5 µM Aβ was 34% and 18% lower compared with the control, respectively, (i.e. 29±3 pN and 36±2 pN for cells treated with respective 1 µM and 0.5 µM Aβ in Fig. 7A). When cells were treated with latrunculin A, similar results to those with Aβ treatment were obtained. Interestingly, when lovastatin was applied prior to treatment with Aβ, *F_mtf_* was similar to that of the control (Fig. 7A).

In order to further investigate the effects of Aβ on *F_mtf_*, histograms of *F_mtf_* measured from different experimental groups (filled bars) were plotted and superimposed with those from the control group (unfilled bars). This comparison showed that in the control group, a distribution of *F_mtf_* with a major population peak at *F_mtf_*≈32.5 pN was obtained (Fig. 7B). Histamine enhanced actin polymerization in cells, and shifted the major population peak to a higher *F_mtf_*≈44 pN, while Latrunculin A disrupted F-actin, resulting in the peak at a lower *F_mtf_*≈22.5 pN (Fig. 7B). Interestingly, cells treated with 0.5 µM of Aβ produced two population peaks at *F_mtf_*≈20 pN and 39 pN (Fig. 7B). The major population peak corresponding to the lower *F_mtf_* produced by Aβ suggests that Aβ caused a disruption of subcellular connectivity between the plasma membrane and cytoskeleton, since the distribution at the lower *F_mtf_* was similar to that of cells treated with Latrunculin A. These mechanical alterations in response to Aβ are dose-dependent, as the peak at the higher *F_mtf_* subsided and only the major peak at the lower *F_mtf_* remained for cells treated with a higher dose of Aβ (1 µM) (Fig. 7B). Pretreatment of cells with lovastatin suppressed these mechanical alterations induced by Aβ, as the *F_mtf_* distribution was almost totally overlapped with that of the control experiment (Fig. 7B).

### Characterization of Cell membrane stiffness

**Figure 8:**
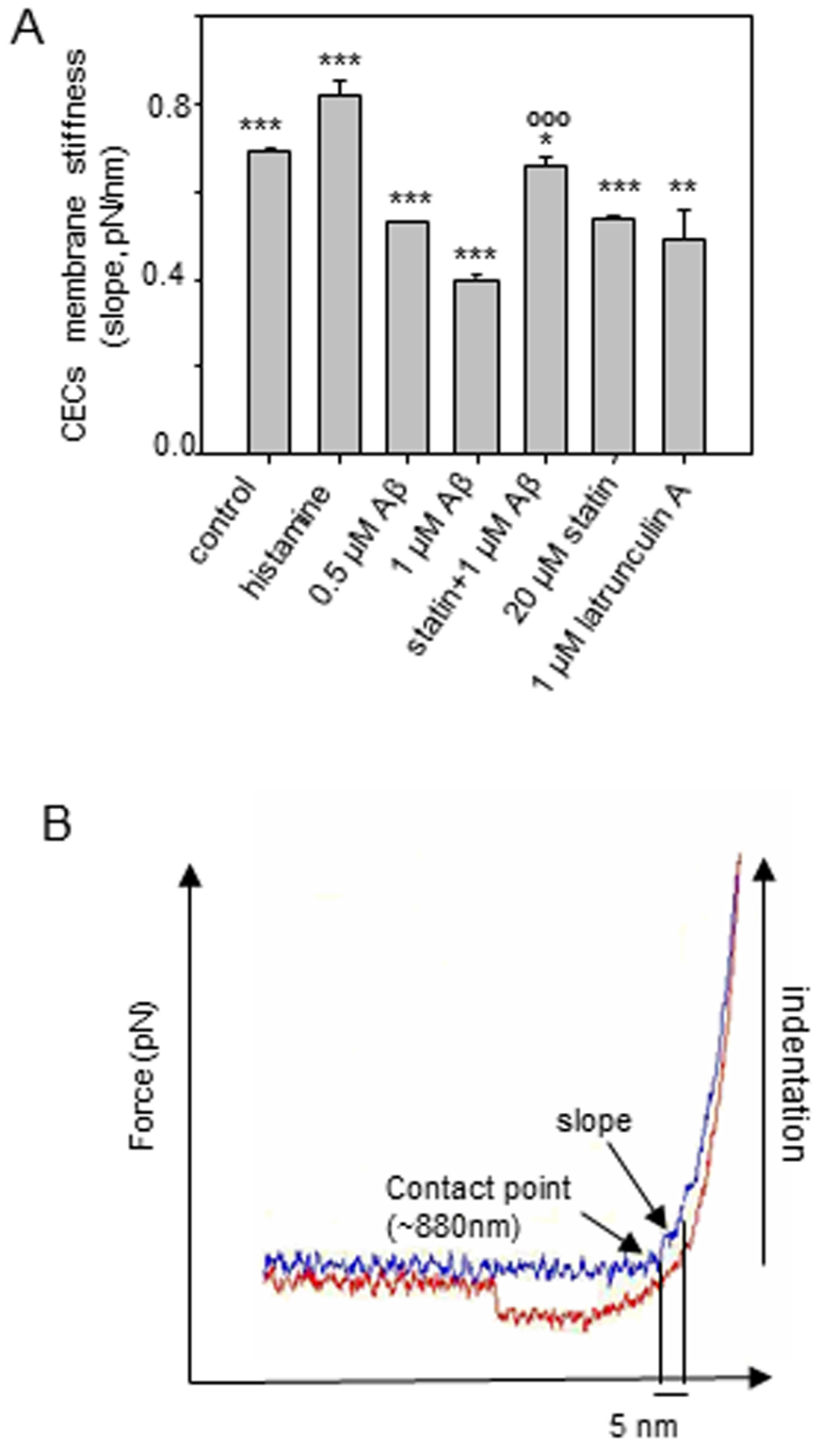
Membrane stiffness. (*A*) Membrane stiffness for cells treated with histamine, Aβ, lovastatin and Aβ, lovastatin alone, and latrunculin A. (*B*) Membrane stiffness is measured by calculating the slope (denoted by the arrow) from 5 nm indentation at the cell surface. ***p≤0.001, **p≤0.01 compare to the control; °°p≤0.01, °°°p≤0.001 compare to the Aβ (1 µM) treatment group.

The *F_mtf_* data suggests that the treatment of CECs with Aβ caused lower connectivity between plasma membranes and cytoskeleton. It is believed that higher cytoskeletal connectivity to membranes should provide additional mechanical strength to plasma membranes; therefore, cell membrane stiffness can be a measure for cytoskeletal connectivity to membranes (i.e. the membrane-cytoskeleton adhesion). To quantify cell membrane stiffness, we analyzed the “approaching” force curves when the cantilever made a ∼5 nm indentation at the cell surface (Fig. 8B). 5 nm is about the thickness of the bilayer membrane. Consistent with data of actin polymerization and *F_mtf_*, treatment with histamine resulted in a higher cell membrane stiffness, while Latrunculin A resulted in a lower cell membrane stiffness. (Fig. 8A) On the other hand, Aβ caused lower cell membrane stiffness (Fig. 8A), suggesting that Aβ produced lower cytoskeletal connectivity to plasma membranes in cells. The lower cell membrane stiffness resulted from Aβ was suppressed by lovastatin (Fig. 8A).

## Discussion

Primary capture of circulating monocytes from a bloodstream mediated by membrane tethering is the initial mechanical step in transmigration. This dynamic process requires special mechanisms for establishing stable cell-cell contact. The P- and E-selectins are the adhesion molecules that specialize in mediating this process on activated endothelium. P-selectin is stored in Weibel-Palade bodies inside the endothelial cells and can be mobilized to the cell surface within minutes [11]. Earlier studies showed upregulation of P-selectin expression in brain ECs associated with enhanced transmigration of immune cells across the BBB in pathological conditions, such as ischemia and atherosclerosis [26], [27]. There is also evidence that Aβ soluble aggregates selectively activate cerebral vascular endothelium and increase transmigration of monocytes across the BBB [6], [7]. Our data here show that Aβ_42_ oligomers promoted the expression of P-selectin at the surface of the CECs. Since E-selectin induction occurs on the transcriptional level, the duration of Aβ treatment (20 min) did not lead to a detectable change of E-selectin expression.

In addition to adhesion molecules, such as selectin, the mechanical properties of the cell membrane are a critical factor influencing the cell-cell adhesion [13]–[15]. Earlier works have confirmed that lower tether extraction force favors rolling. It has been shown that tether formation reduces the adhesion force between the endothelial cells and leukocytes, assisting the formation of new bonds and stabilizing rolling [28]. Enrichment of endothelial cells with cholesterol has been found to increase tether length and reduce force bond, and increase the bond lifetime, which resulted in increased chance of adhesion [14]. We found that Aβ_42_ oligomers decrease *F_mtf_* and increase probability of adhesion. Taken together, our data suggest that Aβ facilitates primary capture of monocytes and rolling adhesion at the brain endothelial cell surface through promoting P-selectin expression, and lowering *F_mtf_*, which favors adhesion.

Membrane tether extraction strongly depends on the F-actin network condition and membrane-cytoskeleton integrity [29], [30]. It has been shown that disruption of the actin cytoskeleton and glycocalyx backbone removal lead to decrease of adhesion energy [31]. Latrunculin A, a well-known inhibitor of actin polymerization, has been reported to decrease the cell's average elastic modulus and adhesion force [32]–[34]. It is also observed that statins can significantly impair F-actin stress fiber formation [35]. Our data are in good agreement with those reported in the literature showing that latrunculin A and lovastatin lower the amount of F-actin in the CECs. Consistent with the QIM results, our AFM study demonstrated that both lovastatin and Latrunculin A resulted in a decrease of cell stiffness, membrane stiffness, and *F_mtf_*. Consistent with those from others showing the ability for histamine to induce cytoskeletal F-actin polymerization and increase the cell adhesion force [15], [36], [37], our AFM study also showed histamine to increase cell stiffening and higher *F_mtf_*.

Although Aβ has been found to increase actin polymerization and cause formation of actin stress fiber [38], [39], Aβ_42_ treatment surprisingly decreased *F_mtf_* mediated by sLe^x^-selectin bonding. Previous studies have demonstrated that variability in tether extraction force could also provide information in membrane-cytoskeleton association: enhanced membrane-cytoskeleton interactions make tethers less homogeneous, and, vice versa, no significant difference is observed in the case of low connectivity [14]. Our force distribution analysis indicated that in contrast to histamine, Aβ_42_ (1 µM), significantly decreased force range variability (Fig. 7B). Therefore, despite that both histamine and Aβ_42_ enhance P-selectin expression, increase probability of adhesion, and promote actin polymerization, their effects on *F_mtf_* are different. Interestingly, two distinct populations of *F_mtf_* were observed for 0.5 µM Aβ_42_ treatment. The presence of two populations of *F_mtf_* and a lower cell membrane stiffness indicate the ability of Aβ_42_ oligomers to weaken locally-subcellular membrane-cytoskeleton association. Taken together, these mechanical results lead to a new hypothesis that Aβ induces dissociation of the adhesion between the cytoskeleton and the lipid bilayer membrane by disrupting the cytoskeletal linkage to plasma membranes or altering the attachment of transmembrane proteins (e.g., cadherins, integrins) to F-actin.

QIM and AFM data have demonstrated that Aβ_42_ oligomers promoted expression of P- selectin, induced stress fibers formation, increased cell stiffness but decrease membrane stiffness, increased the probability of adhesion, and lower *F_mtf_*; and these effects were suppressed by lovastatin. Statins are the inhibitors of hydroxy-3-methylglutaryl coenzyme A reductase (HMG-CoA), an enzyme that catalyzes the cholesterol synthesis in the liver and other tissues [40]. In addition, statins have been demonstrated to have anti-inflammatory effects independent of cholesterol reduction. They attenuate vascular inflammation, upregulate nitric oxide expression in endothelial cells, microglia and monocytes, inhibit leukocytes recruitment to vascular cells, and significantly decrease the migration of monocytes and lymphocytes across the human BBB [41], [42]. Furthermore, statins have been shown to be potentially therapeutic for AD by inhibiting Aβ-stimulated expression of interleukin-1β and reduce the levels of Aβ [43]–[45]. A retrospective epidemiological study recently demonstrated that long-term treatment of hypercholesterolaemic patients with lovastatin, simvastatin, and pravastatin lowered the risk of developing AD [43], [46]–[48]. However, the mechanisms linking Aβ and statins on CEC function remain poorly understood. Our findings showing the effects of Aβ_42_ on the membrane tether adhesion of cerebral endothelial cells, and how lovastatin counteracts these effects provide new insights into the mechanism of neuroinflammation in AD brains, and may offer new approaches for preventive treatment of the disease. This study should prove to provide insights into new therapeutic strategies, since microglial cell activity is a crucial factor in Aβ clearance and immunotherapy for treatment of AD [49], [50].
